# Toxicity and radiation dosimetry studies of the serotonin transporter radioligand [^18^ F]AFM in rats and monkeys

**DOI:** 10.1186/s13550-014-0071-1

**Published:** 2014-12-29

**Authors:** Ya-Yao Huang, Chen-Yi Cheng, Wen-Sheng Huang, Kuo-Hsing Ma, Ta-Wei Tseng, Ta-Kai Chou, Yiyun Huang, Chyng-Yann Shiue

**Affiliations:** PET Center, Department of Nuclear Medicine, Tri-Service General Hospital, 325 Sec. 2, Cheng-Kung Road, Taipei, 114 Taiwan; PET Center, Department of Nuclear Medicine, National Taiwan University Hospital, 7, Chung-Shan S. Road, Taipei, 100 Taiwan; Departments of Medical Research and Nuclear Medicine, Changhua Christian Hospital, 135, Nan-Hsiao Street, Changhua, 500 Taiwan; Department of Biology and Anatomy, National Defense Medical Center, 161 Sec. 6, Min-Chuan East Road, Taipei, 114 Taiwan; PET Center, Department of Diagnostic Radiology, Yale University School of Medicine, New Haven, CT 06520 USA

**Keywords:** [^18^ F]AFM, Serotonin transporter, Toxicity, Radiation dosimetry

## Abstract

**Background:**

[^18^ F]AFM is a potent and promising PET imaging agent for the serotonin transporter. We carried out an acute toxicity study in rats and radiation dosimetry in monkeys before the translation of the tracer to humans.

**Methods:**

Single- and multiple-dose toxicity studies were conducted in Sprague–Dawley rats. Male and female rats were injected intravenously with AFM tartrate as a single dose of 98.7 or 987 μg/kg (592 or 5,920 μg/m^2^, 100× or 1,000× the proposed human dose of 8 μg, respectively) on day 1 or as five consecutive daily doses of 98.7 μg/kg/day (592 μg /m^2^/day, 100× human dose, total dose 493.5 μg/kg). PET/CT scans were performed in four Formosan rock monkeys (two males and two females, each monkey scanned twice) using a Siemens BIOGRAPH scanner. After injection of [^18^ F]AFM (88.5 ± 20.3 MBq), a low-dose CT scan and a series of eight whole-body PET scans in 3-D mode were performed. Time-activity data of source organs were used to calculate the residence times and estimate the absorbed radiation dose using the OLINDA/EXM software.

**Results:**

In the rats, neither the single dose nor the five daily doses of AFM tartrate produced overt adverse effects clinically. In the monkeys, the radiation doses received by most organs ranged between 8.3 and 39.1 μGy/MBq. The osteogenic cells, red marrow, and lungs received the highest doses of 39.1, 35.4, and 35.1 μGy/MBq, respectively. The effective doses extrapolated to male and female adult humans were 18.0 and 18.3 μSv/MBq, respectively.

**Conclusions:**

Toxicity studies in Sprague–Dawley rats and radiation dosimetry studies in Formosa rock monkeys suggest that [^18^ F]AFM is safe for use in human PET imaging studies.

**Trial registration:**

IACUC-12-200.

## Background

Abnormalities in the serotonin transporter (SERT) have been implicated in several neurologic and psychiatric disorders [1,2]. SERT is also the target for selective serotonin reuptake inhibitors (SSRIs), a class of compounds used as antidepressants clinically [3,4]. Thus, *in vivo* SERT imaging in humans would assist in the early diagnosis as well as monitor the efficacy of the treatment in these diseases [5]. The isoquinoline analog [^11^C]-(+)McN5652 was the first PET agent for studying SERT in humans [6-8]. However, this agent has high nonspecific binding and has only moderate signal contrast in human PET studies [9]. In addition, ^18^ F has some advantages over ^11^C, notably that the ^18^ F-labeled radioligands can be transported off-site due to its longer half-life. Thus, two of its fluorine-18-labeled analogs, S-[^18^ F]fluoroethyl)-(+)-McN5652 [10] and S-[^18^ F]fluoromethyl)-(+)-McN5652 [11], have been synthesized and evaluated as SERT imaging agents. The S-[^18^ F]fluoromethyl)-(+)-McN5652, in particular, has recently been shown to be suitable for *in vivo* quantification of SERT with PET in human [12]. In addition to isoquinoline, the diaryl sulfides *N*,*N*-dimethyl-2-(arylthio)benzylamines (403U76) have been reported to possess very high selectivity and affinity for SERT binding sites [13].Thus, [^11^C]3-amino-4-(2-dimethylaminomethyl-phenylsulfanyl)benzonitrile ([^11^C]DASB) [14], [^11^C]2-[2-(dimethylaminomethyl)phenylthio] -5-fluoromethylphenylamine ([^11^C]AFM) [15], [^11^C] *N*,*N*-dimethyl-2-(2-amino-4-methylphenyl-thio)benzylamine ([^11^C]MADAM) [16], and [^11^C]*N*,*N*-dimethyl-2-(2′-amino-4′-hydroxymethyl -phenylthio)benzylamine ([^11^C]HOMADAM) [17] were also synthesized and found to be the potential radioligands for studying SERT in humans [18-21]. The results of these studies showed that all of these carbon-11-labeled diphenyl sulfides were better than [^11^C]-(+)-McN5652 as SERT imaging agents for human studies. Thus, some of their ^18^ F-labeled analogs, such as 2-[(2-amino-4-chloro-5-[^18^ F]fluorophenyl)thio]-*N*,*N-*dimethyl-benzenmethanamine ([^18^ F]-ACF) [22], *N*,*N*-dimethyl-2-(2-amino-4-[^18^ F]fluorophenylthio)benzylamine (4-[^18^ F]-ADAM) [23-25], *N*,*N*-dimethyl-2-(2-amino-5-[^18^ F]fluorophenylthio)-benzylamine (5-[^18^ F]-ADAM) [26], 2-[[2-amino-4-([^18^ F]fluoromethyl)phenyl]thio]-*N*,*N*-dimethylbenzenemethanamine ([^18^ F]AFM) [27], 2-(2′-((dimethylamino)methyl)-4′-(3-[^18^ F]fluoropropoxy)phenylthio)benzenamine ([^18^ F]FPBM) [28], and 3-amino-4-(2-((4-[^18^ F]fluorobenzyl)methylamino)methylphenylsulfanyl)-benzonitrile ([^18^ F]FBASB) [29] have been synthesized and evaluated in animals as potential SERT imaging agents [22,26-33]. Of these ^18^ F-labeled diaryl sulfides, 4-[^18^ F]-ADAM and [^18^ F]AFM have been shown to possess proper characteristics as brain SERT imaging agents for human studies [24,34]. Thus, we have studied the toxicity and radiation dosimetry of 4-[^18^ F]-ADAM in rats and monkeys. The results showed that 4-[^18^ F]-ADAM was safe and suitable for human studies [35], and the results of the distribution of 4-[^18^ F]-ADAM in human brain were reported recently [36]. In order to access the suitability of [^18^ F]AFM as a brain SERT imaging agent for human studies, we report herein the toxicity and radiation dosimetry of [^18^ F]AFM in rats and monkeys before its human studies are undertaken.

## Methods

The protocols used for this study were similar to those used for the study of 4-[^18^ F]-ADAM [35].

### Synthesis of AFM tartrate

The AFM tartrate was synthesized according to the method reported previously [27]. The identity and chemical purity of this compound were confirmed by ^1^H NMR, mass spectroscopy, HPLC analysis, and elemental analysis.

### Toxicity studies

The toxicity studies were performed in male and female Sprague–Dawley rats by the Biosciences Division of the SRI International (SRI), Menlo Park, CA and were fully supported by the NIMH Toxicological Evaluation of Novel Ligands Program.

Animal procedures conducted at SRI were approved by SRI’s Institutional Animal Care and Use Committee (IACUC). SRI’s animal facilities have been accredited by the Association for Assessment and Accreditation of Laboratory Animal Care International (AAALAC) and are registered with the US Department of Agriculture as a Research Facility. SRI files assurances with the Public Health Service (PHS) Office of Laboratory Animal Welfare (OLAW) and adheres to PHS standards and practices.

### Animals

Forty male (6 to 6 1/2 weeks old, weighing 143 to 250 g) and 40 female (7 to 7 1/2 weeks old, weighing 152 to 213 g) Sprague–Dawley rats (Charles River Laboratories, Wilmington, MA, USA) were used in this study. Upon arrival, the rats were placed in quarantine for 3 days. The general appearance of the animals was evaluated by the attending veterinarian, who documented that the animals were healthy before releasing them from quarantine. The rats were fed with Purina Certified Rodent Chow, #5002 (Purina Mills, Richmond, IN, USA) *ad libitum*. Water (purified, reverse osmosis) was provided *ad libitum* during quarantine and study periods. The rats were housed (five per cage) in clear polycarbonate cages in an animal room that was monitored for temperature (70°F to 74°F) and humidity (29% to 58%) in a light cycle of 12 h light/12 h dark.

### Preparation of dose formulation

The test article, AFM tartrate, was formulated in sterile phosphate buffered saline (PBS) (AMRESCO, Inc., Solon, OH, USA). Dose formulations were prepared by dissolving the appropriate amount of test article in the vehicle to achieve target concentrations using a sterile stir bar and sonication. Lower concentrations were achieved by serial dilution from the high dose concentration. Doses were based on the free base (290.49 g/mol; 65.95% of total molecular weight). Dose formulations were stored frozen up to 7 days at −72°C to −76°C until the day of use. Formulations were brought to room temperature prior to administration to the animals. Drugs were administered through intravenous (iv) injection via lateral tail vein. The intravenous route is intended for human clinical trials. Therefore, iv injection was selected to model the intended route of human administration.

### Verification of formulation

All AFM tartrate formulations used for the toxicity studies were analyzed by SRI staff using reversed-phase HPLC methods [Luna C18 analytical column (4.6 × 250 mm, 5 μm particle size); mobile phase: solvent A: 0.1% TFA in water; solvent B: 0.1% TFA in CH_3_CN; gradient: solvent A/solvent B 85/15 for 10 min, 50/50 for 1 min, 85/15 for 4 min; flow rate: 1.0 mL/min; UV detector: 240 nm; ambient temperature]. The HPLC system used was Hewlett-Packard Model 1100 Series liquid chromatography system (Agilent Technologies, Inc., Santa Clara, CA, USA). Data were analyzed using HP Chemstation Software, version A. 06.04. Concentration, homogeneity, and stability of the formulation were confirmed before testing and were found to be within 10% of the target concentrations for all treatment groups.

### Dosage calculation

The procedures used to determine the AFM tartrate dose to be administered to rats required that all doses were converted from units of mg/kg (body weight) to units of body surface area expressed in mg/m^2^. Basing the dose on body surface allows for determination of an equivalent dose to another species. The equation used was:

$$ \mathrm{Dose}\left(\raisebox{1ex}{$ mg$}\!\left/ \!\raisebox{-1ex}{$ kg$}\right.\right)\kern0.5em \times \kern0.5em F\kern0.5em =\kern0.5em \mathrm{Dose}\left(\raisebox{1ex}{$ mg$}\!\left/ \!\raisebox{-1ex}{${m}^2$}\right.\right) $$

where *F* is a constant based on the species of animal being tested (a value of 37 for humans) [37]. Doses were based on the body weight recorded on day 1 before dosing (and converted to mg/m^2^ surface area), and preset multiples of the maximum proposed 8-μg dose to be administered to a 50-kg person were administered to the test dose groups (Table 1). The doses used in these studies, expressed in both mg/kg and mg/m^2^, as well as individual species *F* values can be found in Table 1.

**Table 1:** **Experimental dosage regimens for toxicity study of AFM tartrate in male and female rats**

		**Male**			**Female**		
		**Vehicle**	**1,000× human dosage** ^**a**^	**100× human dosage** ^**a**^	**Vehicle**	**1,000× human dosage** ^**a**^	**100× human dosage** ^**a**^
No. of animals		10	10	10	10	10	10
Dose per day	By surface area (μg/m^2^)	0	5,920	592	0	5,920	592
	By body weight (μg/kg)	0	987	98.7	0	987	98.7
No. dosages		1	1	1/5	1	1	1/5
Total dosage (μg/kg)		0	987	98.7/493.5	0	987	98.7/493.5
Dosage volume (mL/kg)		7.58	7.58	7.58/7.58	7.58	7.58	7.58/7.58
Rats killed^b^							
Interim	Day	3	3	3/7	3	3	3/7
	No. of killed	5	5	5/5	5	5	5/5
Terminal	Day	15	15	15/19	15	15	15/19
	No. of killed	5	5	5/5	5	5	5/5

^a^Maximum human dose is 8 μg per 50-kg person, i.e., 0.16 μg/kg; 0.16 × 37 = 5.92 μg/m^2^. Scaling for the rat gives 5.92 μg/m^2^/6 = 0.987 μg/kg as an equivalent human dosage. 1,000 × 0.987 = 987 μg/kg as the high dosage; ^b^Five rats in each group and of each sex were killed on day 3 (2 days following single dosing) and five on day 15 (14 days following single dosing). Five rats in each group and of each sex were killed on day 7 (2 days following 5-day dosing) and five on day 19 (14 days following 5-day dosing). Dosing of the 5-day dosing animals was initiated 4 days prior to dosing of the single-dose animals so that necropsies occurred on the same calendar day for all groups, thus allowing sharing of control clinical pathology and necropsy data between the two dosage regimens.

### Dosing procedure

Male and female rats (10/sex/gp) were given a single iv dose of AFM tartrate at 98.7 or 987 μg/kg (592 or 5,920 μg/m^2^, 100× or 1,000× the proposed human dose of 8 μg, respectively) on day 1 or daily iv dose on days 1 to 5 for five consecutive days at 98.7 μg/kg/day (592 μg/m^2^/day, 100× human dose, total dose of 493.5 μg/kg). A control group (10/sex) was given a single iv dose of vehicle, 1% ascorbic acid in sterile phosphate buffered saline, at an equivalent volume on day 1. Dose injection of the repeat dose group was initiated 4 days prior to dosing of the single dose animals so that necropsies occur on the same calendar day for all groups, thus allowing for sharing of control clinical pathology and necropsy data between the two dose regimens.

### Observation protocol

All animals in this study were observed at least once daily for signs of mortality, morbidity, injury, and availability of food and water. Detailed clinical observations were recorded daily throughout the study. Individual body weights were measured and recorded for each animal on day 1 prior to dosing and at necropsy (days 3 and 15 for groups 1 to 3 and days 7 and 19 for group 4). Food consumption was measured for an approximately 24-h period twice weekly throughout the study. Blood for clinical pathology evaluation was collected on days 3 and 15 (interim and terminal necropsy of groups 1 to 3) or days 7 and 19 (interim and terminal necropsy of group 4). Animals were sacrificed by overdose with sodium pentobarbital (150 mg/kg) administered by intraperitoneal injection.

### Clinical pathology

After collection at SRI, the clinical pathology samples were sent to Quality Clinical Labs, Inc (Mountain View, CA, USA). Blood samples were collected into tubes containing ethylenediaminetetraacetic acid (EDTA) (hematology samples), sodium citrate (coagulation samples), or no anticoagulant (serum chemistry samples).

### Gross necropsy

On days 3 and 15 (interim and terminal necropsy of groups 1 to 3) or days 7 and 19 (interim and terminal necropsy of group 4), after recording of body weight and collection of clinical pathology blood samples, animals were sacrificed and given a complete *postmortem* examination, which included a thorough inspection of all body orifices and surfaces and an examination of all cranial, thoracic, and abdominal organs. Organ weights were measured for adrenal glands, brain, heart, kidneys, liver, spleen, thymus, testes, and ovaries. All of the organs and tissues were retained and fixed in phosphate buffered 10% formalin.

### Histopathologic examination

Sections of the retained tissues were embedded in paraffin, cut approximately 5-μm thick, and stained with hematoxylin and eosin by Environmental Pathology Laboratories, Inc. (Sterling, VA, USA). All tissues from the animals in the control and treated groups were examined by a board-certified veterinary pathologist. If a tissue had gross findings at the time of necropsy, the same tissue from the other dosage groups was also examined.

### Statistical analyses

One-way ANOVA was performed for body weight, food consumption, organ weights, and clinical pathology data (LABCAT® modules: In-Life v. 6.2, HE v.4.42, and OW v. 3.24). When appropriate, a *post hoc* analysis (Dunnett’s *t*-test) was carried out. In all cases, the lower limit for statistical significance was defined as *p* ≦ 0.05.

### Dosimetry studies of [^18^ F]AFM in monkeys

#### Synthesis of [^18^ F]AFM

**Scheme 1:**
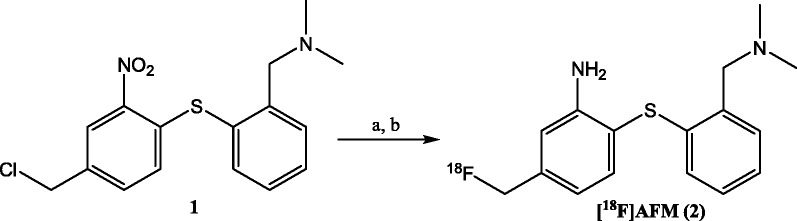
**Synthesis of [** ^**18**^ **F]AFM (2). (a)** K[18 F]/K_2.2.2_, DMSO/*t*-BuOH, 85°C; **(b)** NaBH_4_-Cu(OAc)_2_, EtOH, 80°C.

The radioligand [^18^ F]AFM (**2**) was synthesized by the reported method (Scheme 1) [27] except using an automated synthesizer as previously described for the synthesis of 4-[^18^ F]-ADAM [25]. Briefly, nucleophilic fluorination of 2-[2-(dimethylaminomethyl)phenylthio]-5-chloromethylphenylamine (**1**) (6.6 mg in 0.1 mL of DMSO and 0.4 mL of *t*-BuOH) with dried potassium [^18^ F]fluoride/Kryptofix_2.2.2_ at 85°C for 15 min followed by reduction with Cu(OAc)_2_ (21 mg in 1 mL of EtOH) and NaBH_4_ (8.2 mg in 0.6 mL of EtOH) at 80°C for 20 min and purification with HPLC (EP250/16 Nucleosil 100–7 C18 column, 16 × 250 mm, 10 μm, Macherey-Nagel, MN, USA); Mobile phase: CH_3_CN:0.1 M HCO_2_NH_4_ (30:70 containing 0.3 vol.% acetic acid); flow rate: 10 mL/min. The HPLC product fraction was diluted with excess of H_2_O (250 mL) and passed through a tC_18_ cartridge into the waste bottle. The tC_18_ cartridge was rinsed with an additional 10 mL of H_2_O into the waste bottle. The [^18^ F]AFM retained in tC_18_ cartridge was rinsed out with 1 mL of EtOH into a vial which contained 10 mL of normal saline and was then passed through a Millipore filter (Cathivex GS, 0.22 mm; Millipore, Billerica, MA, USA) into a sterile vial. The formulated solution contains less than 10% EtOH in normal saline. The radiochemical yield of [^18^ F]AFM was 0.6% ± 0.1% (EOS) (*n* = 8) in a synthesis time of 100 min from EOB. The radiochemical purity of the product was greater than 98% with a specific activity of 62.9 ± 18.5 GBq/μmol (1.7 ± 0.5 Ci/μmol) (*n* = 8). TLC (Silica gel/CH_3_OH:CH_2_Cl_2_ (1:9), Rf = 0.55) and HPLC (C18 column: Phenomonex Luna (2), 5 μm, 4.6 × 250 mm; mobile phase: CH_3_CN:0.1 M HCO_2_NH_4_ (30:70 with 0.3 vol.% acetic acid)); flow rate: 1 mL/min; Rt = 10.5 min showed that [^18^ F]AFM was not contaminated with [^18^ F]fluoride and was stable for more than 4 h in the formulated solution.

#### Animals

The animal study protocol used for this study was approved by the IACUC of the National Defense Medical College. Four Formosan rock monkeys (*Macaca cyclopis*), two females, 2.70 and 2.85 kg, respectively, and two males, 5.78 and 7.25 kg, respectively, were used in the dosimetry study. Animals were fasted overnight and immobilized with ketamine (2 mg/kg), anesthetized with 2% isoflurane via an endotracheal tube, and administered with atropine sulfate (2 mg i.m.) to minimize secretions during the course of the experiment. Body temperature was kept constant at 37°C with a heated blanket. The heart rates *p*O_2_ and *p*CO_2_ were checked every 10 min and kept in the normal range throughout the imaging sessions.

#### PET imaging protocol

Whole-body transmission and emission scans were acquired with a Biograph PET/CT scanner (Biograph Duo, Siemens, Knoxville, TN, USA), which has 58.5-cm transverse field-of-view (FOV), 15.5-cm axial FOV, and 4.8-mm FWHM spatial resolution. After [^18^ F]AFM (88.5 ± 20.3 MBq, 2.4 ± 0.5 mCi; *n* = 8) injection, a low dose CT scan (130 kVp, 50 mAs) and a series of eight whole-body PET scans were performed (15, 55, 95, 110, 155, 170, 215, and 230 min, respectively, post-injection). Total acquisition time was 240 min. Each scan covered the monkey’s body from the head to the thigh and consisted of five or six bed positions depending on the size of the monkey. In each bed position, data were acquired for 2 min in 3-D mode. The data were then reconstructed by Ordered-Subsets Expectation-Maximization (OSEM) on a 128 × 128 matrix (slice thickness, 5 mm), with two iterations and eight subsets, 3-mm FWHM gaussian filter, and corrected by photon attenuation using the CT scan.

#### Image analysis

For all organs except bone, the image analysis and residence time calculations were performed by the reported methods with some modifications [35,38]. Each CT and PET whole-body images of [^18^ F]AFM were successively loaded to the PMOD 2.5 software (PMOD technologies, Zurich, Switzerland; www.pmod.com) to generate the fusion images, and the organs were identified and outlined. Regions of interest (ROIs) of these organs were manually drawn as precisely as possible on the organ itself on each horizontal slice. The bone uptake of [^18^ F]AFM was determined by the bone ROI that was drawn in the forearm over the radii and ulnae (left and right) on the transmission scan and then applied to the corresponding PET image. Because the bone marrow is primarily located in the ribs, vertebrae, and pelvis but not in the distal limbs [39], the uptake of [^18^ F]AFM in the bone marrow was derived from ROIs of the lumbar vertebrae. The activity in each organ was non-decay-corrected and expressed as percent-injected dose per organ (*n* = 8, mean ± SD).

#### Residence times and absorbed dose calculations

To calculate the radiation dose of each organ as well as the effective dose, the injected dose of [^18^ F]AFM and the non-decay-corrected time-activity curves (TACs) of the brain, lungs, heart, liver, spleen, kidneys, radii, ulnae, lumbar vertebrae, and bladder of the four monkeys were individually entered into an Excel spreadsheet and the data processed individually for each animal. The difference between the injected dose and the sum of the whole-body radioactivity (assuming that there was no excretion) plus the radioactivity in the above-mentioned organs was taken as the TAC of the remainder.

The residence time (*h*) of the selected organ was calculated as the area under the TAC of the source organ from time 0 to infinity over the initial total body activity (about 5 half-lives of ^18^ F, i.e., about 550 min). The area under the TAC of each organ was generated using the following strategy including trapezoidal integration of the first four TAC data points through the origin and exponential decline of the four remaining TAC data points to infinity such that the residence times of these organs were obtained [40].

The residence time (*h*) of bone and the bone marrow were estimated using the reported method [38]. Briefly, since the bone mass of the ulna and radius equals 4.3% of all bone mass in the body [41], the residence time of all bones in the body was calculated as that of the radii and ulnae divided by 4.3%.

The residence time of all red marrow in the body was estimated from the lumbar vertebrae, which contains both red marrow and bone. Since the mass of the red marrow in lumbar vertebrae equals 12.3% of the mass of all red marrow in the body [42], the residence time of the red marrow in the body was calculated as that of all radioactivity in the lumbar vertebrae minus that from the bone component and then divided by 12.3%.

The human dosimetry was estimated from both male and female monkey biodistribution data. Except for the bone and red marrow, the organ weight and body mass were used for allometric scaling [43]. That is, the residence time in each organ was converted to the corresponding human value by multiplication with a factor to scale organ and body weights (in kilograms) as (*w*_m,b_/*w*_m,o_)(*w*_h,o_/*w*_h,b_), where *w*_m,b_ was the monkey body weight, *w*_m,o_ was the monkey organ weight, *w*_h,b_ was the human body weight, and *w*_h,o_ was the human organ weight. However, the *w*_m,o_ values for the Formosan rock monkeys were unavailable, so instead, we used the *w*_m,o_ values for the rhesus monkey (*Macaca mulatta*) in our calculations. Both monkey organ weights [44] and human organ weights [45] were obtained from the literatures. Monkey bone and red marrow were assumed to have the same percentage of total body weight as that for human. The residence time of [^18^ F]AFM in the rest of the body was obtained by subtracting the total organ residence time from the reciprocal of the ^18^ F decay constant.

Absorbed radiation doses were calculated from the residence times in all source organs for each monkey by entering the information into the Java-based OLINDA 1.0/EXM computer program [46] using the model for a 70-kg adult male and female phantom.

## Results

### Toxicity studies of AFM in rats

The toxicity of AFM was studied in rats using single dosing and multiple dosing paradigms. The following parameters were evaluated: mortality/morbidity, clinical observations, body weights, food consumption, clinical pathology (hematology and serum chemistry), organ weights, gross necropsy observation, and microscopic histopathologic observations. The results showed that a single iv dosing of AFM tartrate at 987 μg/kg (1,000× human dosage) or repeated iv dosing at 98.7 μg/kg/day (100× human dosage) for 5 consecutive days did not produce overt adverse effects clinically. Thus, AFM presents minimal toxicity, and its no observable adverse effect level (NOAEL) is considered to be greater than 987 μg/kg for a single i.v. dose administration and greater than 98.7 μg/kg/day for a 5-day repeated i.v. dosing regimen.

### Biodistribution of [^18^ F]AFM in monkeys

**Figure 1:**
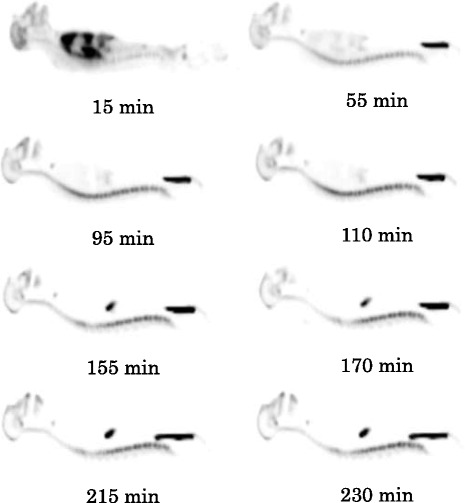
**Typical sagittal view of [** ^**18**^ **F]AFM whole-body images of a male Formosan rock monkey at different time points.**

Typical whole-body biodistribution of [^18^ F]AFM in a Formosan rock monkey was depicted in Figure 1. The brain, heart, liver, spleen, lungs, kidneys, bladder, and lumbar vertebrae were visually identified on the images and treated as source organs of radioactivity for dosimetry calculation of [^18^ F]AFM in monkeys. The uptakes of [^18^ F] AFM in selected organs are shown in Table 2 and expressed as percentage injected dose per organ (%ID/organ, non-decay-corrected). The uptakes of [^18^ F]AFM in the lungs, liver, brain, and heart were high in the early time points and declined slowly for the brain, whereas the radioactivity declined rapidly in the lungs, liver, and heart. On the other hand, the uptakes of [^18^ F]AFM in kidneys, bladder, red marrow, and bone were low in the beginning and gradually increased throughout the experiments. However, there appeared to be significant difference in the uptake of bladder among individual monkeys. The uptake of radioactivity in red marrow is difficult to calculate because it is located within the bones, and ^18^ F-labelled radioligands might be metabolized through de-fluorination, leading to bone uptake of [^18^ F]fluoride ion. Based on the reported method [38], we estimated that the uptake of radioactivity in all red marrow of the adult was approximately 4.7% of the injected activity at 230 min.

**Table 2:** **Biodistribution and residence times of [** ^**18**^ **F]AFM in Formosan rock monkeys** ^**a**^

	**%ID/organ**								
	**Brain**	**Lungs**	**Heart**	**Liver**	**Spleen**	**Kidneys**	**Bone**	**Red marrow**	**Bladder**
Time (min) post-injection									
15	2.24 ± 0.69	19.26 ± 4.98	1.77 ± 0.27	3.94 ± 0.87	0.17 ± 0.05	2.09 ± 0.53	7.53 ± 2.71	3.40 ± 0.51	0.07 ± 0.04
55	1.98 ± 0.43	5.43 ± 1.42	0.79 ± 0.16	3.03 ± 0.46	0.07 ± 0.02	1.26 ± 0.46	11.17 ± 4.35	7.48 ± 1.46	0.62 ± 0.92
95	1.36 ± 0.30	2.73 ± 0.54	0.43 ± 0.07	2.21 ± 0.43	0.04 ± 0.02	0.74 ± 0.33	9.08 ± 4.36	8.03 ± 2.13	0.78 ± 1.04
110	1.18 ± 0.27	2.21 ± 0.39	0.36 ± 0.05	1.98 ± 0.42	0.03 ± 0.01	0.63 ± 0.30	8.70 ± 3.97	7.89 ± 2.20	0.82 ± 1.13
155	0.81 ± 0.20	1.35 ± 0.22	0.22 ± 0.03	1.30 ± 0.48	0.02 ± 0.01	0.41 ± 0.22	7.21 ± 3.23	6.69 ± 2.15	0.67 ± 1.05
170	0.72 ± 0.17	1.18 ± 0.17	0.19 ± 0.02	1.17 ± 0.46	0.02 ± 0.01	0.35 ± 0.20	6.34 ± 3.12	6.34 ± 2.13	0.66 ± 1.10
215	0.44 ± 0.21	0.72 ± 0.29	0.11 ± 0.05	0.79 ± 0.36	0.01 ± 0.01	0.20 ± 0.15	4.47 ± 2.44	5.16 ± 1.49	0.47 ± 0.68
230	0.39 ± 0.19	0.64 ± 0.25	0.10 ± 0.04	0.70 ± 0.34	0.01 ± 0.01	0.17 ± 0.13	4.09 ± 2.42	4.51 ± 1.19	0.42 ± 0.61
Residence time (*h*)	0.051 ± 0.013	0.153 ± 0.045	0.034 ± 0.011	0.069 ± 0.025	0.035 ± 0.002	0.019 ± 0.002	0.069 ± 0.031^b^	0.325 ± 0.080	0.018 ± 0.024^d^
							0.277 ± 0.125^c^		

^a^ *n* = 8, mean ± SD; non-decay-corrected; ^b^Residence time for trabecular bone; ^c^Residence time for cortical bone; ^d^Residence time for subjects 1 to 3, 4 to 5, and 6 to 7 were 0.0418 ± 0.0247, 0.0059 ± 0.0037, and 0.0007 ± 0.0002, respectively.

### Absorbed radiation dose estimates

The residence times of selected organs in monkeys are also listed in Table 2. Among these organs, red marrow, bone, lungs, liver, and brain had long residence times. However, the evaluation of residence time in the bladder showed larger variations because of the discrepant biodistribution of [^18^ F]AFM in the bladder among individual monkeys. Using the above residence times, the individual organ doses in humans extrapolated from the male and female monkeys were calculated with OLINDA/EXM software, and the results are shown in Table 3. The osteogenic cells received the highest dose, followed by red marrow, lungs, heart, kidneys, bladder, and liver. The effective doses in male and female humans, according to ICRP 60 [47] and extrapolated from the data in male and female monkeys, were 20.2 and 20.6 μSv/MBq, respectively.

**Table 3:** **Radiation dosimetry estimates for [** ^**18**^ **F]AFM using the means of four male and four female monkeys**

	**Male**	**Female**	**Total**
Target organ			
Adrenals^a^	14.0 ± 0.1	16.8 ± 0.4	15.1 ± 1.4
Brain^a^	12.8 ± 2.2	12.4 ± 1.2	12.7 ± 1.8
Breasts^a^	9.0 ± 0.5	10.5 ± 0.5	9.5 ± 0.9
Gallbladder^a^	12.6 ± 0.3	13.9 ± 0.8	13.1 ± 0.8
LLI wall^a^	12.4 ± 0.8	15.2 ± 0.8	13.5 ± 1.6
Small intestine^a^	12.4 ± 0.6	14.0 ± 0.8	13.0 ± 1.0
Stomach^a^	11.3 ± 0.6	13.7 ± 0.8	12.2 ± 1.4
ULI wall^a^	11.9 ± 0.6	14.5 ± 0.8	12.9 ± 1.4
Heart wall^a^	21.0 ± 1.6	19.3 ± 1.0	20.3 ± 1.6
Kidneys^a^	18.9 ± 1.3	21.8 ± 0.8	20.0 ± 1.9
Liver^a^	15.7 ± 2.7	14.7 ± 1.8	15.3 ± 2.3
Lungs^a^	36.6 ± 6.3	32.6 ± 4.6	35.1 ± 5.8
Muscle^a^	10.0 ± 0.4	12.4 ± 0.5	10.9 ± 1.3
Ovaries^a^	12.8 ± 0.8	15.2 ± 0.8	13.7 ± 1.4
Pancrease^a^	13.1 ± 0.3	15.4 ± 0.7	14.0 ± 1.2
Red marrow^a^	35.8 ± 4.8	34.7 ± 6.5	35.4 ± 5.1
Osteogenic cells^a^	32.8 ± 4.8	49.5 ± 5.7	39.1 ± 9.8
Skin^a^	7.6 ± 0.3	9.5 ± 0.4	8.3 ± 1.0
Spleen^a^	11.0 ± 1.9	12.0 ± 0.6	11.4 ± 1.6
Testes^a^	9.0 ± 0.8	-	9.0 ± 0.8
Thymus^a^	11.5 ± 0.5	13.5 ± 0.6	12.3 ± 1.1
Thyroid^a^	10.3 ± 0.5	12.1 ± 0.5	11.0 ± 1.0
Urinary bladder^a^	23.1 ± 12.9	11.1 ± 0.7	18.6 ± 11.6
Uterus^a^	12.6 ± 1.3	14.3 ± 0.8	13.3 ± 1.4
Total body^a^	12.2 ± 0.1	15.3 ± 0.0	13.4 ± 1.6
Effective dose equivalent (EDE)^b^	20.2 ± 0.5	20.6 ± 0.5	20.3 ± 0.5
Effective dose (ED)^b^	18.0 ± 0.5	18.3 ± 0.4	18.2 ± 0.5

Means of four male and four female monkeys (*n* = 8, mean ± SD) extrapolated to 70 kg adult male and female humans. ^a^In μGy/MBq; ^b^In μSv/MBq.

## Discussion

[^18^ F]AFM has been demonstrated to be a promising SERT imaging agent in rats and baboons [27,34]. In order to advance it as a potential SERT imaging agent in humans, it is necessary to estimate its toxicity profile and the organ radiation dose burden in animal models.

Toxicity studies of AFM were carried out in rats to determine potential toxic effects, identify potential target organs of toxicity, and determine its NOAEL. Since some patients may undergo [^18^ F]AFM PET imaging more than once during a short period of time (e.g., within 1 or 2 weeks), we used both single and multiple dosing paradigms in the rat toxicity study. The dosages administered to rats were 100 to 1,000 times more than the proposed mass dose to be administered to humans. The results showed that intravenous administration of AFM tartrate to rats for a single day at 98.7 or 987 μg/kg (592 or 5,920 μg/m^2^, 100× or 1,000× the proposed human dose of 8 μg, respectively) or 5 consecutive days at 98.7 μg/kg/day (592 μg/m^2^/day, 100× human dose, total dose of 493.5 μg/kg) did not produce overt adverse effects clinically nor had any detectable toxicity on any target organ. Although the maximum tolerated dose (MTD) of AFM could not be determined from this study, it is believed to be greater than 987 μg/kg for a single iv dose administration and 98.7 μg/kg/day for 5 consecutive days. Thus, the NOAEL is considered to be greater than 987 μg/kg for a single iv dose administration and greater than 98.7 μg/kg/day for a 5-day repeated iv dosing regimen which is similar to those of 4-F-ADAM (1,023.7 μg/kg for a single iv dose and greater than 102.37 μg/kg/day for a 5-day repeated iv dosing regimen) [35]. Whole-body distribution study of [^18^ F]AFM was carried out in Formosan rock monkeys. Following [^18^ F]AFM injection, the initial uptakes of radioactivity in the brain, lungs, heart, and liver were high. The radioactivity then washed out from the brain slowly while it cleared from the lungs, heart, and liver rapidly (Table 2). The fact that the uptake of [^18^ F]AFM in the lungs was high (19.3%ID/organ at 15 min post-injection) was also observed for other SERT radioligands, such as [^11^C](+)McN5652, [^11^C]DASB, [^123^I]ADAM, and 4-[^18^ F]-ADAM ([35] and references cited therein). The exact reason(s) for the high uptake of these radioligands in the lungs was unclear. It may be due to the specific binding of the circulating serotonin on pulmonary membranes, nonspecific binding of amine by the macrophage, and a large blood volume in the lungs ([35] and references cited therein), or it may be due to the abundance of SERT expression in the lungs, with concentration higher than that in the brain [48]. The radioactivity in the bladder increased with time suggesting [^18^ F]AFM may be eliminated via the same hepato-billiary and renal pathways as that of [^11^C]DASB, [^123^I]ADAM, and 4-[^18^ F]-ADAM ([35] and references cited therein).

**Figure 2:**
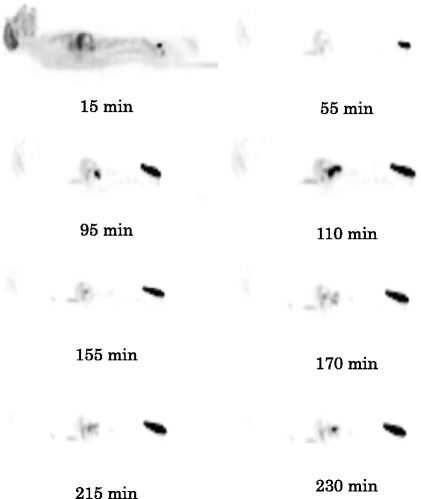
**Typical sagittal view of 4-[** ^**18**^ **F]-ADAM whole-body images of a male Formosan rock monkey at different time points.**

**Figure 3:**
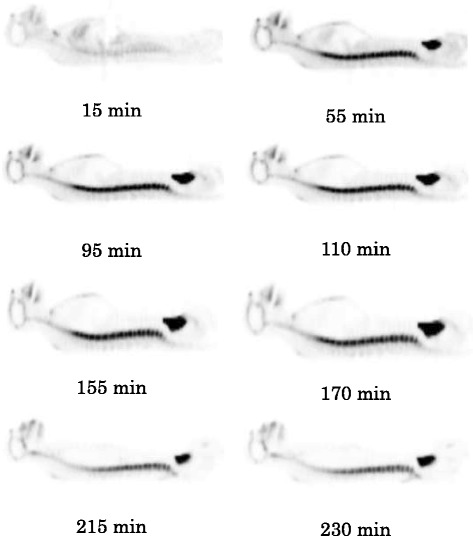
**Typical sagittal view of [** ^**18**^ **F]NaF whole-body images of a male Formosan rock monkey at different time points.**

SERT are expressed in the brain as well as in various peripheral organs and tissues such as the adrenals, stomach, gut, spleen, kidneys, lungs, heart, uterus, and bone marrow [49,50]. Following [^18^ F]AFM injection, the uptake of radioactivity in the spine was high (Figure 1). In contrast, the uptake of radioactivity in the spine was relatively low following 4-[^18^ F]-ADAM injection (Figure 2). If the uptake of radioactivity in the spine is solely due to the binding of [^18^ F]AFM to SERT in the bone marrow within the spine, then it should also have a similar uptake of 4-[^18^ F]-ADAM in this region. The fact that the uptake of [^18^ F]AFM in the spine was relatively high compared to that of 4-[^18^ F]-ADAM (Figure 2) and that its whole body distribution in monkeys was somewhat similar to that of [^18^ F]NaF (Figure 3) suggests that [^18^ F]AFM may de-fluorinate *in vivo,* leading to uptake of the free [^18^ F]fluoride in the bone of the spine region. This has also been observed in another ^18^ F-labeled SERT imaging agent, [^18^ F](+)-FMe-McN5652 in rats and humans [12,51]. It should be pointed out, though, that it may be tempting to infer that *in vivo* defluorination occurs with [^18^ F]AFM in monkeys, as the ^18^ F label is located at the benzylic position of the molecule and presumed to be labile. However, it is worth noting that while we were careful to choose regions (radii and ulnae and lumbar vertebrae) with minimal confounding sources of radioactivity, the method we used to estimate the radioactivity in these regions (spine, bone, and red marrow) is vulnerable to extrapolation error, because these regions represent a small portion of the total bone or bone marrow as indicated by Terry et al*.* [38]. Therefore, at the present, it is reasonable to assume that uptake in the spine region reflects the combined contribution of [^18^ F]AFM binding to SERT in the spinal cord [52,53] as well as uptake in the marrow and bone structure. A direct comparison of the whole-body distribution of [^18^ F]AFM and [^11^C]AFM in monkeys probably will clarify the issue whether the high uptake of [^18^ F]AFM in the spine region is solely due to [^18^ F]fluoride ion, its binding to SERT or in combination of both.

The guidelines on radiation exposure for human subjects involved in research studies varied internationally. Radiation risk estimates were recently issued by the National Institutes of Health (NIH) in the United States, and the effective dose-estimates are considered to be the most accurate measure of radiation risks. Based on the NIH guidelines, the maximum exposure is 50 mSv of effective dose per year for a research subject [54]. On the other hand, under the guidelines of the European Commission, the intermediate risk levels in adults will be equivalent to an effective dose range of 1 to 10 mSv per annum [55]. To estimate the human dosimetry from the whole-body PET images data in Formosan rock monkeys, the organ weight and body mass were used for allometric scaling after obtaining the residence time of each organ for each subject. Since the organ weights of the Formosan rock monkeys are unavailable and the rhesus monkeys (*Macaca mulatta*) are genetically similar to the Formosan rock monkeys [56,57], we used the organ weights of the rhesus monkey (*Macaca mulatta*) to calculate the human dosimetry, similar to what we have done for 4-[^18^ F]-ADAM [35]. The results (Table 3) show that the estimated human dosimetry of [^18^ F]AFM is 18.21 μSv/MBq which is comparable to 4-[^18^ F]-ADAM (19.3 μSv/MBq) and those of other SERT, DAT, or NET imaging agents ([35] and references cited therein). The critical organs are the osteogenic cells, red marrow, and lungs. The lungs are also reported to be one of the critical organs for other SERT imaging agents such as [^11^C]DASB [40,58] and 4-[^18^ F]-ADAM [35]. However, compared to other widely used ^18^ F-radiopharmaceuticals, such as [^18^ F]FDG (119 μGy/MBq) [59] and [^18^ F]FDOPA (159 μGy/MBq) [60], the radiation doses of [^18^ F]AFM in critical organs are relatively low.

## Conclusions

The toxicity studies in rats showed that a single iv dosing of AFM tartrate at 987 μg/kg (1,000× human dosage) or repeated iv dosing at 98.7 μg/kg/day (100× human dosage) for 5 consecutive days did not produce overt adverse effects clinically. Thus, AFM presents minimal toxicity, and its NOAEL is considered to be greater than 987 μg/kg for a single iv dose administration and greater than 98.7 μg/kg/day for a 5-day repeated iv dosing regimen.

The radiation dosimetry estimates obtained from Formosa rock monkeys suggest that the received doses of most organs range between 8.3 and 39.1 μGy/MBq. The osteogenic cells, red marrow, and lungs are considered to be the critical organs with radiation dose burdens of 39.1, 35.4, and 35.1 μGy/MBq, respectively, for male and female adults, which are all below NIH guidelines. These results indicate that [^18^ F]AFM appears to be safe for human studies from both pharmacologic and radiation exposure perspectives. However, the fact that [^18^ F]AFM has relatively low radiochemical yield may hinder its broad use as a SERT radioligand and a more efficient, high-yield radiosynthetic method may need to be developed before its advance to PET imaging applications in humans.

## Abbreviations

FOV: field-of-view
SERT: serotonin transporter
SSRIs: selective serotonin reuptake inhibitors
SRI: SRI International
IACUC: Institutional Animal Care and Use Committee
PHS: public health service
OLAW: Office of Laboratory Animal Welfare
PBS: phosphate buffered saline
MTD: maximum tolerated dose
OSEM: ordered-subsets expectation-maximization
ROIs: regions of interest
TACs: time-activity curves
NOAEL: no observable adverse effect level
NIH: National Institutes of Health.
