# Erratum to: Toxicity and radiation dosimetry studies of the serotonin transporter radioligand [^18^F]AFM in rats and monkeys

**DOI:** 10.1186/s13550-016-0222-7

**Published:** 2016-09-06

**Authors:** Ya-Yao Huang, Cheng-Yi Cheng, Wen-Sheng Huang, Kuo-Hsing Ma, Ta-Wei Tseng, Ta-Kai Chou, Yiyun Huang, Chyng-Yann Shiue

**Affiliations:** 1 PET Center, Department of Nuclear Medicine, Tri-Service General Hospital, National Defense Medical Center, 325 Sec. 2, Cheng-Kung Road, Taipei, 114 Taiwan; 2 PET Center, Department of Nuclear Medicine, National Taiwan University Hospital, 7, Chung-Shan S. Road, Taipei, 100 Taiwan; 3 Departments of Medical Research and Nuclear Medicine, Changhua Christian Hospital, 135, Nan-Hsiao Street, Changhua, 500 Taiwan; 4 Department of Biology and Anatomy, National Defense Medical Center, 161 Sec. 6, Min-Chuan East Road, Taipei, 114 Taiwan; 5 PET Center, Department of Diagnostic Radiology, Yale University School of Medicine, New Haven, CT 06520 USA

## Erratum

Unfortunately, the original version of this article was published with incorrect details for one authors name and address.

Cheng-Yi Cheng’s name, which had been incorrectly spelled as Chen-Yi Cheng, has been corrected. The author’s correct address is as follows:

PET Center, Department of Nuclear Medicine, Tri-Service General Hospital, National Defense Medical Center, 325 Sec. 2, Cheng-Kung Road, Taipei 114, Taiwan.
